# Higher Self-Control Capacity Predicts Lower Anxiety-Impaired Cognition during Math Examinations

**DOI:** 10.3389/fpsyg.2016.00485

**Published:** 2016-03-31

**Authors:** Alex Bertrams, Roy F. Baumeister, Chris Englert

**Affiliations:** ^1^Department of Educational Psychology, Institute of Educational Science, University of BernBern, Switzerland; ^2^Department of Psychology, Florida State UniversityTallahassee, FL, USA

**Keywords:** anxiety, math performance, self-control, self-efficacy, self-esteem

## Abstract

We assumed that self-control capacity, self-efficacy, and self-esteem would enable students to keep attentional control during tests. Therefore, we hypothesized that the three personality traits would be negatively related to anxiety-impaired cognition during math examinations. Secondary school students (*N* = 158) completed measures of self-control capacity, self-efficacy, and self-esteem at the beginning of the school year. Five months later, anxiety-impaired cognition during math examinations was assessed. Higher self-control capacity, but neither self-efficacy nor self-esteem, predicted lower anxiety-impaired cognition 5 months later, over and above baseline anxiety-impaired cognition. Moreover, self-control capacity was indirectly related to math grades via anxiety-impaired cognition. The findings suggest that improving self-control capacity may enable students to deal with anxiety-related problems during school tests.

## Introduction

Successful performance on tests and examinations is an important determinant of long-term life outcomes in modern society. It is therefore hardly surprising that many people suffer from anxiety about test performance—anxiety that can cause various unwelcome and problematic impairments (Sarason, 1959; Spielberger and Vagg, 1995). The present investigation tested hypotheses about three personality processes that held out some promise of enabling people to overcome anxiety problems. Specifically, we examined the potential impact of dispositional self-control capacity, self-efficacy, and self-esteem on anxiety-related problems.

### Anxiety and cognitive performance

*Anxiety* can generally be understood as an unpleasant emotional experience. Spielberger (1983) distinguished between state anxiety (i.e., heightened arousal, feelings of tension and nervousness, and worrisome thoughts in an ongoing situation) and trait anxiety (i.e., the general, dispositional tendency to experience anxiety in threatening situations). *Test anxiety* has been conceptualized as an aversive emotional arousal that occurs specifically in connection with evaluative situations, especially academic exams (Spielberger and Vagg, 1995; Zeidner, 1998). Test anxiety itself can be differentiated from its detrimental consequences for cognition during tests (Eysenck et al., 2007). Researchers from various lines of research such as on test anxiety (e.g., Wine, 1971), math anxiety (e.g., Ashcraft and Kirk, 2001), choking under pressure (e.g., Beilock et al., 2004), stereotype threat (e.g., Schmader and Johns, 2003), and achievement motivation (e.g., Elliot and McGregor, 1999) generally agree that experiencing threat or anxiety during tests may distort information processing, thereby impairing performance on the test. Anxiety appears able to interfere with all stages of information processing, so that anxious people suffer impairments in encoding, storing, organizing, elaborating upon, and retrieving information (see Zeidner, 1998, for an overview).

One way that anxiety degrades information processing is that it causes test-takers to divert attention to task-irrelevant matters, especially worries about their performance and the consequences of failure. Building on that insight, Eysenck et al. (2007) proposed Attentional Control Theory. It argues that anxious worries preoccupy the central executive of the working memory system (Baddeley, 1997). As a result, behavior tends to be guided more by external stimuli and the stimulus-driven attentional system—and correspondingly less by the goal-directed attentional system (Corbetta and Shulman, 2002). Hence, anxious persons suffer from an automatic tendency to pay less attention to answering the test questions and more attention to distracting factors that pop up either in the environment or in their own minds, such as worrying about the potential consequences of failing the test (see also Bar-Haim et al., 2007).

The key point is that test anxiety impairs concentration and other processes needed for optimal performance. For math tests, these other processes would include quantitative reasoning and calculating. These effects were the primary focus of our investigation. We shall refer to the detrimental effects of anxiety on concentration, quantitative reasoning, and other cognitive processes as anxiety-impaired cognition (see Rost and Schermer, 1989, 2007). Previous work has linked anxiety-impaired cognition to lower test grades, particularly in math tests (Sparfeldt et al., 2005; Rost and Schermer, 2007).

Not all anxious people suffer cognitive impairments to the same degree (Rost and Schermer, 1989, 2007; see also Schilling et al., 2004). That is, in different people, the same degree of anxiety produces different degrees of problems with concentrating, thinking, understanding, following instructions, memory, and catching one's own mistakes, all while taking tests. To understand these differences, it is useful to invoke another aspect of Eysenck et al.'s (2007) Attentional Control Theory, which is that anxious people are motivated to compensate for the detrimental effects of anxiety on performance. They are often but differentially able to accomplish this by increasing effort and by using auxiliary resources (see also earlier theorizing of Eysenck and Calvo, 1992). The present research sought to predict this differential success by considering three potential such resources. The next three sections will detail these hypotheses.

### Self-control

Our primary hypothesis was that self-control would help mitigate anxiety-impaired cognition. Self-control is the process of overriding or altering one's dominant response tendencies (Muraven and Baumeister, 2000; Baumeister et al., 2007). In particular, self-control is used to overcome affective, cognitive, and behavioral tendencies that could otherwise prevent people from achieving their goals (Baumeister et al., 2007). Attentional control is an important form of self-control (Luszczynska et al., 2004). As Schmeichel and Baumeister (2010) explained, attentional control helps people to focus on task-relevant information and screen out task-irrelevant information. In that way, attentional self-control enables people to avoid distractions and thereby to focus on what is most relevant and important. Applied to the situation of a math test, attentional self-control should enable people to keep distracting worries and other anxiety-related thoughts at bay, so that one can concentrate on solving the test problems. Because attentional control is a form of self-control, we hypothesized that people with poor self-control would suffer more anxiety-related problems than people with good self-control (see also Englert and Bertrams, 2015).

If self-control is all it takes to prevent anxiety from impairing cognition and lowering math grades, why do some people fail to exert self-control? One answer is that self-control appears to depend on a limited energy resource. According to the strength model of self-control, energy is depleted when one exerts effortful control to alter one's responses (Baumeister et al., 2007). In the depleted state, the person seeks to conserve energy, so self-regulatory performance suffers (Muraven et al., 2006). A person may be temporarily or chronically low in such energy, and in that case the person may balk at expending more energy to combat the negative anxiety effects.

Ample evidence has already indicated that individual differences in self-control affect adjustment, performance, and behavior, including in school settings (e.g., Mischel et al., 1989; Tangney et al., 2004; Finkenauer et al., 2005; Kuhnle et al., 2010, 2012; Oertig et al., 2013). Much of this work has used the Trait Self-Control Scale (Tangney et al., 2004; for a meta-analysis, see de Ridder et al., 2012). However, we wanted to use a measure more specifically concerned with the availability of energy resources for self-control. Ciarocco et al. (2007) developed a self-report measure of current state of self-regulatory resources. We adapted this (using the German version by Bertrams et al., 2011) to measure trait availability of these resources. To be sure, adapting a state measure to use as a trait measure contains some risk that it will not be stable, so we measured it twice in order to be able to calculate retest reliability.

### Self-efficacy

*Perceived self-efficacy* is the degree to which people believe they are capable of doing what is needed for success. Bandura (1986) defined it as “people's judgments of their capabilities to organize and execute courses of action required to attain designated types of performances” (p. 391). Efficacy expectations help people to deal successfully with stressful and threatening situations, partly because they guide effort to overcome obstacles and withstand aversive circumstances (Bandura, 1977). For a test-anxious person, a math test would be precisely such a stressful and threatening situation. Hence we reasoned that high self-efficacy would help people to reduce patterns of anxiety-impaired cognition.

Previous work has provided some evidence to support the prediction that high self-efficacy would help people to put more effort into overcoming effects of anxiety during tests. Sarason et al. (1986) showed that higher self-efficacy was associated with lower cognitive interference from task-irrelevant worries during task performance. Karademas et al. (2007) found that higher levels of self-efficacy led to paying less attention to threat-related stimuli (i.e., less attentional bias). These findings indicate that self-efficacy is positively related to the ability to exert attentional control.

### Self-esteem

*Self-esteem* is the (typically global) appraisal of one's self-worth and positive qualities (e.g., James, 1890). It is one of the most widely studied concepts in psychology (Judge, 2009). Abundant evidence has correlated high self-esteem with better grades in school (e.g., Wylie, 1979), although some have concluded that self-esteem is the result rather than the cause of school performance (Baumeister et al., 2003).

Nonetheless, on an a priori basis, one could expect that high self-esteem would be useful for combating effects of anxiety. Self-esteem has been found to serve as a valuable buffer against effects of anxiety (Pyszczynksi et al., 2004). Moreover, some evidence indicates that high self-esteem facilitates mobilizing effort in threatening test situations (Perez, 1973; Shrauger and Sorman, 1977; Tafarodi and Vu, 1997). In that sense, self-esteem is highly relevant to Eysenck et al.'s Attentional Control Theory, because high self-esteem should help people exert themselves to focus attention on doing well on the test and correspondingly to minimize anxiety-impaired cognition.

For sure, there is some overlap among self-control, self-efficacy, and self-esteem (e.g., Tangney et al., 2004; Finkenauer et al., 2005; Luszczynska et al., 2005; Bertrams, 2012). Our procedures therefore sought to establish independent effects as well as to disentangle which variables were mainly responsible for the effects.

## Methods

### Participants

The sample consisted of 158 secondary school students (103 female; mean age at baseline = 17.97 years, *SD* = 0.96) from a vocational business school in southern Germany. Informed consent was obtained in advance.

### Statistical power

In experiments, self-control capacity has typically a medium-to-large effect on self-control dependent measures (*d* = 0.62; see Hagger et al.'s, 2010 meta-analysis). Given the relatively large time lag time between the assessment of self-control capacity (predictor) and anxiety-impaired cognition (dependent variable) in the present study, we expected a smaller effect (i.e., *f*^2^ = 0.07). A power analysis using G^*^Power 3.1 (Faul et al., 2007) indicated that a sample size of at least 115 participants would be required to detect an effect of this size (with α = 0.05, 1−β = 0.80). With *N* = 158 the present sample was thus well-powered.

### Materials

Except for math grades, all variables were measured with reliable and validated multiple-item scales. The inner consistencies for the applied measures in the present study are displayed in Table 1.

**Table 1:** **Descriptive statistics and correlations**.

**Variable**	**α**	***M*(*SD*)**	**Correlations**									
			**1**	**2**	**3**	**4**	**5**	**6**	**7**	**8**	**9**	**10**
1. Anxiety-impaired cognition (T1)	0.90	2.66 (0.96)	−									
2. Anxiety-impaired cognition (T2)	0.93	2.73 (0.92)	0.58^***^	−								
3. Self-control capacity (T1)	0.88	3.01 (0.39)	−0.35^***^	−0.31^***^	−							
4. Self-control capacity (T2)	0.92	2.87 (0.46)	−0.31^***^	−0.45^***^	0.69^***^	−						
5. Self-efficacy (T1)	0.83	2.89 (0.46)	−0.27^***^	−0.10	0.50^***^	0.27^***^	−					
6. Self-esteem (T1)	0.83	3.19 (0.51)	−0.30^***^	−0.17^*^	0.59^***^	0.37^***^	0.59^***^	−				
7. Test anxiety (T1)	0.89	2.59 (0.70)	0.37^***^	0.35^***^	−0.44^***^	−0.34^***^	−0.28^***^	−0.43^***^	−			
8. Test anxiety (T2)	0.92	2.63 (0.75)	0.18^*^	0.48^***^	−0.26^**^	−0.43^***^	−0.11	−0.30^***^	0.60^***^	−		
9. Math grade (T1)^a^	−	4.05 (1.32)	−0.19^*^	−0.35^***^	0.01	0.13	−0.15	−0.08	−0.28^***^	−0.36^***^	−	
10. Math grade (T2)^a^	−	4.00 (1.21)	−0.30^***^	−0.43^***^	0.08	0.18^*^	−0.06	−0.03	−0.19^*^	−0.34^***^	0.56^***^	−
11. Gender^b^	−	−	−0.08	−0.14	0.12	0.16^*^	0.16^*^	0.17^*^	−0.20^*^	−0.27^***^	0.08	0.07

*N = 158. Overall scores of a psychometric scale were obtained by averaging the responses to the scale items. T1 = first time of measurement (i.e., baseline measure in September 2011), T2 = second time of measurement (i.e., February 2012)*.

a In Germany the best possible grade is 1 and the worst is 6. To ease the presentation, we recoded the grades such that higher values represent better math performance.

b *Coding: 1 = female, 2 = male*.

* p < 0.05, two-tailed.

** p < 0.01, two-tailed.

*** *p < 0.001, two-tailed*.

#### Anxiety-impaired cognition

Our primary focus was on how strongly anxiety interfered with cognition during math tests. To measure this, we used the eight-item subscale *anxiety-impaired cognition* (German: Kognitive Angstmanifestation) from the Differential Test Anxiety Inventory (German: Differentielles Leistungsangstinventar; Rost and Schermer, 1989, 2007). The scale was designed for use with German-speaking students. We used the brief version of the scale, which the authors developed for research purposes. The scale was preceded by its original instructions; however, in line with previous research, we added the specification that responses were to pertain specifically to mathematics tests (see Sparfeldt et al., 2005).

In detail, the students were informed that the questionnaire will assess how anxiety occurs in students during test situations in the subject math. First, they were asked to think back about negative personal experiences with test situations in the subject math. Then the instructions emphasized that the issue is not whether anxiety is experienced frequently or rarely, but how intensely different reactions are experienced *when one is anxious*. The item stem for each item was “When I am anxious in a test situation in the subject math….” The items (e.g., “…my thoughts are blocked”) were answered on 5-point scales labeled with *very weak intensity* (1), *somewhat weak intensity* (2), *moderate intensity* (3), *somewhat strong intensity* (4), and *very strong intensity* (5). The scale has proven to be reliable and valid in previous research (Schilling et al., 2004; Sparfeldt et al., 2005; Rost and Schermer, 2007). Recent research using latent state-trait theory modeling suggests that the situational specificity of the measurement occasion does not affect responding to math-related anxiety measures (Jenßen et al., 2015).

#### Self-control capacity

To assess students' self-control capacity as individual differences variable, we used a 25-item scale developed by Bertrams and Englert (2013). This scale was originally adapted from the German version of the State Self-Control Capacity Scale (English original: Ciarocco et al., 2007; Bertrams et al., 2011), a state measure that has been shown to be sensitive to situational fluctuations in self-control capacity. In the present study, the students were asked to choose for each of the statements the answer that applied to them in general. A sample item is “When I am tempted by something, it is very difficult to resist” (reversely coded). Participants made their responses on a 4-point scale with the labels *almost never* (1), *sometimes* (2), *often* (3), and *almost always* (4).

We administered the self-control capacity scale on both times of measurement, in order to ascertain its test-retest reliability. As can be seen in Table 1, the measure demonstrated satisfactory test-retest reliability over a period of 5 months, as well as high inner consistencies at both times of measurement. Thus, the scale was reliable and depicted relatively stable individual differences. Moreover, prior research (Bertrams and Englert, 2013) and further pretesting yielded evidence for the convergent and divergent validity of the self-control measure^1^.

#### Self-efficacy

For the assessment of self-efficacy, we used the German version of the Generalized Self-Efficacy Scale (Schwarzer and Jerusalem, 1995). The students responded to the 10 items (e.g., “I can usually handle whatever comes my way”) on 4-point scales. The points were labeled with *not at all true* (1), *hardly true* (2), *moderately true* (3), and *exactly true* (4).

#### Self-esteem

The students completed the German version of the Rosenberg (1965), Self-Esteem Scale (Collani and Herzberg, 2003). The 10 items (e.g., “I am able to do things as well as most other people”) were answered on 4-point scales with points labeled as *not at all true* (1), *hardly true* (2), *moderately true* (3), and *exactly true* (4).

#### Test anxiety

As we will explain in detail below, test anxiety was assessed as a control measure. For this purpose, we used the brief version of the Test Anxiety Inventory-German (Wacker et al., 2008). Participants reported their typical experiences during test situations on nine items, using scales labeled *almost never* (1), *sometimes* (2), *often* (3), and *almost always* (4). A sample items is “My heart is pounding.”

#### Math grades

We asked the students to indicate their last report card math grade. Students' self-reports of their report card math grades have been found to strongly correspond to teachers' reports of the same grades (Dickhäuser and Plenter, 2005: *N* = 866 German secondary school students; *r* = 0.88; *r*_*corr*_ = 0.92; time lag between students' reception of the report cards and self-report of the respective report card math grades was 4.5 months). At the first time of measurement, the students reported the math grades from the final report card of the past school year, whereas at the second time of measurement, the reported grades referred to the recently received intermediate report card of the ongoing school year. In Germany, grades vary between 1 (*very good*) and 6 (*insufficient*). For the sake of easier readability, we recoded the grades as such that higher values represent higher math performance.

### Procedure

In September 2011 (henceforth T1), briefly after the beginning of the school year in this part of Germany, the participating students completed a questionnaire that contained the measures of anxiety-impaired cognition during math tests (baseline), self-control capacity, self-efficacy, self-esteem, test anxiety, and math grade in the last report card. The second time of measurement took place 5 months later, in February 2012 (henceforth T2), briefly after the students had received their intermediate report card. The students reported their new report card math grades, and again their experience of anxiety-impaired cognition during math tests. They also reported again their self-control capacity (in order to check for test-retest reliability) and their test anxiety. The questionnaires included further measures that were intended for other research purposes and not of relevance in the present study.

## Results

### Dealing with missing values

There was a maximum of three missing values (1.9%) on any item. We applied the expectation-maximization algorithm using the available data set to estimate and impute these missing values (Dempster et al., 1977). Therefore, all analyses are based on the entire sample of 158 participants.

### Correlation analyses

Table 1 provides descriptive statistics and bivariate correlations for the reported measures. Self-control capacity, self-efficacy, and self-esteem overlapped considerably at T1, *r*s > 0.50, *p*s < 0.001. Therefore, bivariate correlations do not reflect to what extent each of these variables explained unique variance in anxiety-impaired cognition at T2. The next section will analyze independent contributions with multiple regression analysis.

Nonetheless, the simple correlations did provide useful information. Self-control capacity was significantly correlated with anxiety-impaired cognition (the latter measured 5 months later), *r* = −0.31, *p* < 0.001. Self-esteem also significantly predicted anxiety-impaired cognition, although more weakly, *r* = −0.17, *p* = 0.04. The effect of self-efficacy was in the predicted direction but fell short of significance, *r* = −0.10, *p* = 0.20. The self-control correlation was significantly stronger than the self-esteem one (that is, −0.31 differed from −0.17), *z* = 2.00, *p* = 0.045 (two-tailed) by Fisher's test. Self-control also predicted anxiety-impaired cognition more strongly than self-efficacy did (−0.31 vs. −0.10), *z* = 2.71, *p* = 0.007.

We note, too, that self-control capacity was measured twice. The second measure was closer in time to the anxiety-impaired cognition measure and would therefore in a sense furnish the most relevant test of the hypothesis. It yielded an even higher estimate than the T1 numbers reported in the previous paragraph: T2 self-control predicted T2 anxiety-impaired cognition at *r* = −0.45, *p* < 0.001. T2 self-control was also the only one of the personality trait measures to yield a significant predictor of math grades (also T2), *r* = 0.18, *p* = 0.03.

Several of the correlations were relevant to subsequent analyses. Test anxiety was correlated with both anxiety-impaired cognition and self-control capacity. Therefore, we supplemented the multiple regression analysis with an additional analysis including test anxiety as covariate (see below: Auxiliary Analyses). This was done to ensure that the proposed relationship between anxiety-impaired cognition and self-control capacity would not be attributable to their joint overlap with test anxiety.

Anxiety-impaired cognition had a significant negative correlation with math grades on both measurement occasions, indicating its detrimental impact on performance. Neither T1 self-control capacity, nor T1 self-efficacy, nor T1 self-esteem had a direct relationship with math grades at T1 or T2. T1 self-control yielded a trend in the predicted direction, whereas for self-esteem and self-efficacy, the nonsignificant trends were in the opposite (negative) direction. Therefore, math performance was not considered as a control variable in the multiple regression model. It did however remain plausible that the personality traits could be linked to math performance indirectly, mediated via anxiety-impaired cognition (Rucker et al., 2011). Auxiliary analyses will test these.

Gender was unrelated to the dependent variables (i.e., anxiety-impaired cognition and math grades). Therefore, it was not considered any further.

### Multiple regression analysis

In order to examine the unique contributions of self-control capacity, self-efficacy, and self-esteem to shaping anxiety-impaired cognition during math tests, we regressed anxiety-impaired cognition at T2 on these three predictors in a multiple regression model. We held anxiety-impaired cognition at T1 constant by including it as predictor. The Analysis 1 column of Table 2 shows the standardized regression coefficients (beta weights) at a glance. Self-control capacity at T1 negatively predicted anxiety-impaired cognition at T2 over and above the T1 measures of anxiety-impaired cognition, self-efficacy, and self-esteem, *B* = −0.49, *SE B* = 0.20, *t* = −2.45, *p* = 0.02. Thus, consistent with our hypotheses, higher self-control capacity was linked to lesser anxiety-impaired cognition 5 months later—and this was independent of any overlapping variance with self-efficacy or self-esteem. Self-efficacy, *B* = 0.24, *SE B* = 0.17, *t* = 1.47, *p* = 0.14, and self-esteem, *B* = 0.10, *SE B* = 0.16, *t* = 0.62, *p* = 0.54, were not significant predictors, indicating that they had no unique influence on anxiety-impaired cognition. Indeed, and perhaps surprisingly, the nonsignificant trends suggested that if self-esteem and self-efficacy had any effect, it was in the direction opposite to what was predicted (i.e., high self-esteem and high self-efficacy predicted more rather than less trouble with anxiety).

**Table 2:** **Multiple regression analysis predicting anxiety-impaired cognition at T2 without (Analysis 1) and with (Analysis 2) controlling for test anxiety**.

**Predictor**	**Analysis 1**	**Analysis 2**
Anxiety-impaired cognition (T1)	0.56^***^	0.55^***^
Self-control capacity (T1)	−0.21^*^	−0.17^*^
Self-efficacy (T1)	0.12	0.06
Self-esteem (T1)	0.05	0.15
Test anxiety (T1)		−0.12
Test anxiety (T2)		0.46^***^
Model *F* (*df*)	22.20^***^ (4, 153)	27.10^***^ (6, 151)
Model $R_{\text{adj}}^{2}$	0.35	0.50

*N = 158. T1 = first time of measurement (i.e., baseline measure in September 2011), T2 = second time of measurement (i.e., February 2012). Displayed are standardized regression coefficients (beta weights)*.

* *p < 0.05, two-tailed*.

*** *p < 0.001, two-tailed*.

### Auxiliary analyses: possible confounds and stringent tests

As noted above, test anxiety was positively correlated with anxiety-impaired cognition. The latter is understood as a consequence of anxiety, and so it became possible that our main findings were really about test anxiety rather than anxiety-impaired cognition. To address this potential confound, we repeated the multiple regression analysis described in the preceding section, now adding test anxiety at T1 and T2 as covariates to the model. Table 2 provides the results of this analysis in the Analysis 2 column. As can be seen there, all results of our main analysis (see the Analyses 1 column) remained constant. This was also the case when we controlled only for test anxiety at T1 or at T2. Thus, the findings cannot be attributed to individual differences in test anxiety.

Five items of our self-control capacity measure referred to concentration (e.g., “I feel sharp and focused”). A particularly stringent test of our assumption that self-control capacity predicts anxiety-based impaired information processing would be to delete those items and see whether the remainder of the self-control scale could still predict anxiety-impaired cognition. We built an overall score of self-control capacity with the 20 remaining items (Cronbachs's α = 0.85) and repeated both multiple regression analyses that are shown in Table 2. The results did not change. As in the main analysis, self-control capacity predicted anxiety-impaired cognition, *p*s < 0.04, whereas self-efficacy and self-esteem were not significant predictors and even tended to be associated with increases rather than reductions in anxiety-impaired cognition, *p*s > 0.06.

As already reported, we did not find that T1 trait measures predicted math grades, although T2 self-control capacity did significantly predict T2 math grade. But T1 self-control capacity did predict anxiety-impaired cognition, which in turn predicted poorer math performance (see Table 1). Hence we tested the hypothesis that initial self-control capacity would have an indirect relationship to math performance, mediated by anxiety-impaired cognition during math tests. For this purpose we applied bootstrapping with 5000 resamples using Hayes's (2013) regression based tool PROCESS. In addition to self-control capacity at T1 as independent variable, anxiety-impaired cognition at T2 as mediator, and math grades at T2 as dependent variable, we entered the T1 baseline values of anxiety-impaired cognition and math grades as covariates. The bias-corrected bootstrap 95% confidence interval (see Hayes and Scharkow, 2013) did not include zero, 95% CI [0.01, 0.24], meaning that the indirect effect of self-control capacity on math grades via anxiety-impaired cognition was significantly different from zero at α = 0.05.

Parallel analyses replacing self-control capacity with self-efficacy and then with self-esteem yielded no significant indirect effects, as the confidence intervals included zero. Thus, neither self-esteem nor self-efficacy had any indirect link to grades, mediated by anxiety-impaired cognition.

## Discussion

Effective test-taking is a vital step toward educational and occupational success. Many people fail to perform up to the level of their abilities because anxiety interferes with their cognitive processes, such as by impairing concentration. These impairments are costly to both parties. Test takers end up with poorer performance outcomes than they deserve or could have achieved. Testing organizations do not get the actual diagnostic information which are the purpose of testing. Our study examined three personality traits, each of which was assumed to help people to prevent anxiety from impairing their cognitive processes.

### Summary of main findings

We derived predictions that high self-control capacity, high self-esteem, and high self-efficacy would each help students to minimize anxiety-impaired cognition during math tests. In our study, German secondary school students were tracked across 5 months, and we found evidence that those with high self-control capacity suffered less than others with lower self-control capacity from anxiety-impaired cognition. This effect was specific to self-control and remained significant after controlling for other variables. High self-control capacity also predicted better math grades on the measures that were most closely linked in time (T2). And high self-control had an indirect effect on grades, because anxiety-impaired cognition led to poorer grades, and self-control reduced proneness to anxiety impaired cognition. The indirect mediation relationship was significant (i.e., from self-control to anxiety-impaired cognition to math grades).

Neither self-esteem nor self-efficacy yielded any significant benefits in this sample. In fact, the trends were in the opposite direction of our hypotheses. That is, if there was any sign of possible impact, it was such that people with low self-esteem and low self-efficacy were better at preventing anxiety-impaired cognition. The same was true for the effects on math grades: There were no statistically significant relationships, and all trends indicated that students with low self-esteem and low self-efficacy earned better grades than their peers with higher scores on those traits. No significant indirect mediation effects were found for these variables either.

The only significant direct link from self-control, self-esteem, and self-efficacy to grades was the T2 self-control capacity measure (to T2 math grades). In this, self-control had an advantage, as it was the only one of the three to be measured at the second time, which was closest in time to the grade measure. Still, it does not seem likely that administering the self-esteem or self-efficacy measure at T2 would have furnished a significant result in line with the hypotheses. The T1 measures yielded trends in the opposite direction. In contrast, T1 self-control yielded a fairly strong trend in the predicted direction in relation to T2 grades, and so it is not surprising that reducing the time gap with the T2 measure enabled this to cross into significance.

Our findings were not due to differences in test anxiety, because the results remained largely the same when we controlled for test anxiety. They were not due to some overlap in the questions measuring anxiety-impaired cognition and self-control capacity, because we found the same results after eliminating the self-control items referring to cognitive control (i.e., ability to concentrate). Thus, our findings are best interpreted as indicating that high self-control capacity helps to prevent anxiety from impairing cognition during math tests.

### Implications

Clearly, a math test is one kind of situation in which efficient information processing is vital for success. Therefore, it is hardly surprising that anxiety-impaired cognition was associated with lower grades. Our study examined which factors reduced anxiety-impaired cognition. To our knowledge, this is the first evidence of a causal chain running from having a high capacity for self-control, through reductions in anxiety-impaired cognition, to higher math grades. Even though our initial measure of self-control capacity failed to furnish a significant direct impact on math grades (although our second measure did), the indirect mediation findings showed that the initial (T1) measure still contributed to explaining why some students ended up with better grades (see Rucker et al., 2011).

The present findings fit well with the results of recent laboratory experiments. As Bertrams et al. (2013) found, experimentally manipulated self-control capacity determined whether highly anxious undergraduates underperformed in cognitive tests. These results suggested that students can use self-control to keep anxious worries from impairing their ability to perform well on laboratory tests. The present findings extend the generalizability of these previous studies as we were able to demonstrate that high self-control capacity reduced the degree to which students suffered from anxiety-impaired cognition on actual math tests in a real school setting. Convergence between laboratory and field studies bolsters confidence in these conclusions.

An important novel aspect of the present work was that we were able to distinguish the functional role of self-control capacity from the potential influence of two of the self's other resources. Self-efficacy and self-esteem have long been regarded as powerfully relevant to functional affect, cognition, and behavior (Bandura, 1977; Baumeister et al., 2003). However, we did not find indications that any of these variables was helpful for overcoming anxiety-impaired cognition over and above self-control capacity. In contrast, having high self-control capacity was effective at reducing anxiety-impaired cognition even after its overlap with self-efficacy and self-esteem was statistically removed.

More broadly, then, thinking and feeling positively about oneself does not appear to be sufficient to enable people to manage anxiety effectively during math tests. Instead, regarding oneself as having a good capacity for self-control emerged as the most important and helpful factor.

Previous research has documented the adaptive benefits of self-control for health, social life, and achievement (for a review, see Tangney et al., 2004). The present study provides further evidence that self-control is crucial for success in life. Self-control capacity may furnish anxious people a buffer against information processing failure, thereby indirectly improving their test outcomes and related report card grades. People's grades affect their entire lives. For example, grades are often consulted as useful information when organizations select individuals for university admissions, grants, and employment. Students may benefit from interventions for the improvement of self-control capacity (Baumeister et al., 2006; Friese et al., 2011; Bertrams and Schmeichel, 2013). We agree with Duckworth and Seligman (2005) who claimed that programs aiming at building self-discipline may be the royal road to building academic achievement.

In contrast, our results do not provide evidence for the widely accepted view that enhancing positive self-evaluations is a central key toward helping people to overcome psychological problems and dysfunctional responses. The self-esteem movement in particular has seen considerable effort aimed at raising people's self-esteem (and sometimes self-efficacy; see Haney and Durlak, 1998; Baumeister et al., 2003; van Dinther et al., 2011; Morton and Montgomery, 2013). We do think that self-efficacy and self-esteem contribute positively to happiness, satisfaction, and quality of life, but in the present study they failed to offer any benefits for coping with math anxiety. Hence, the policy implication of findings such as ours is that student performance may benefit more from cultivating self-control than from enhancing self-esteem or self-efficacy. Indeed, if self-control can improve performance outcomes, it may in turn boost self-efficacy and self-esteem as positive side effects.

In the present sample, self-control capacity, self-efficacy, and self-esteem were substantially intercorrelated, and that suggests they are likely to be interrelated in other samples too. As a theoretical implication, self-oriented positive expectations and feelings should therefore be carefully differentiated from self-control capacities in future research. It may be problematic to confound these concepts as some self-efficacy measures have done (see Bandura, 2006). In our data, all three variables yielded significant correlations with anxiety-impaired cognition—but the regression analysis that separated their independent effects found that only self-control was a true predictor. The seemingly positive effects of self-efficacy and self-esteem on anxiety-impaired cognition were thus apparently due to the overlap of those variables with self-control. In order to draw valid conclusions about the role of positive self-evaluations for any kind of outcome, one should carefully take into account their potential overlap with self-control capacity. For instance, a recent study by Bertrams (2012) found that self-efficacy *negatively* predicted subsequent math test performance when its overlap with self-control and related variables was statistically removed.

At first glance, our results question a well-established view on how self-efficacy develops over time. In the present study, self-efficacy was unrelated (with a tendency to be negatively related) to a crucial math performance measure (i.e., report card grade). This finding seems to contradict the notion that mastery experiences are the most influential source of self-efficacy (Bandura, 1986). Consistent with the theory, one would have expected self-efficacy and recent math performance, as measured at the first time of measurement, to be positively related. However, Usher and Pajares (2008) pointed out that “it is unwise to use actual performance measures as an assessment of mastery experience” (p. 782). Students' interpretations of a given grade can substantially differ. A grade that damages one student's self-efficacy may boost another one's, depending on their attitudes or expectations. Given this subjective aspect with respect to school grades, our results should not be seen as evidence against the important role of mastery experiences for the development of self-efficacy.

### Limitations

Despite the longitudinal design, causality can only cautiously be inferred from the present data. Strictly speaking, causality can only be attested based on experimental manipulations. Our work addressed actual student performance in the classroom, and it would have been impractical and unethical to manipulate self-efficacy, self-esteem, and self-control capacity. Hence it is important to complement field studies with laboratory experiments.

Another limitation is inherent in self-report measures, on which the present investigation relied. Implicit measures of self-control and self-evaluation are now available and have been found to have predictive value that is distinct from and independent of their explicit counterparts (e.g., Fishbach and Shah, 2006; Gebauer et al., 2008; Peetz et al., 2014). Future research may profitably investigate the contribution of implicit responses to how people deal with anxiety.

## Concluding remarks

Anxiety has been shown to impair test performance, thus sometimes degrading the validity of the test results and possibly damaging students' ability to qualify for life opportunities that might suit them well. Math tests appear to be here to stay, as does the associated anxiety, and so it is highly desirable to find ways to enable people to perform up to their abilities despite anxiety. The present findings suggest that promoting self-control capacity may contribute more to that desirable outcome than boosting self-esteem or self-efficacy.

## Author contributions

AB substantially contributed to study design. AB, RB, and CE contributed to the writing of the manuscript. All authors approve the final version of the manuscript. The authors agree to be accountable for all aspects of the work in ensuring that questions related to the accuracy or integrity of any part of the work are appropriately investigated and resolved.

### Conflict of interest statement

The authors declare that the research was conducted in the absence of any commercial or financial relationships that could be construed as a potential conflict of interest.

## Footnotes

^1^Prior to the present study, we tested crucial aspects of discriminant validity of our measure of self-control capacity. For theoretical and empirical reasons, we assumed that self-control capacity and subjective vitality would be highly related (Muraven et al., 2008). However, both variables should be separable; for instance, in terms of their relation to perceived stress (Ciarocco et al., 2007). Seventy-nine participants completed the Self-Control Capacity Scale (α = 0.90), the trait version of the Subjective Vitality sCALE (Ryan and Frederick, 1997; α = 0.90), and the Perceived Stress Scale (Cohen et al., 1983; α = 84). As expected, self-control capacity and subjective vitality were strongly correlated, *r* = 0.69, *p* < 0.001. Both variables were also correlated with stress (self-control strength: *r* = −0.65, *p* < 0.001, subjective vitality: *r* = −0.48, *p* < 0.001). In a multiple regression analysis we regressed perceived stress simultaneously on self-control capacity and subjective vitality. Higher self-control capacity predicted lower stress over and above subjective vitality, β = −0.61, *p* < 0.001, whereas subjective vitality was not predictive of stress, β = −0.05, *p* = 0.66. Furthermore, we pretested whether the Self-Control Capacity Scale would predict perceived stress over and above trait self-control (Tangney et al., 2004). By this, we aimed to determine whether the two self-control concepts could be distinguished. While our measure refers to the availability of limited self-control resources, trait self-control refers to proactively shaping and selecting ones environment in order to minimize self-regulatory demands (Hofmann et al., 2012). Eighty-three participants completed the Self-Control Capacity Scale (α = 0.92), the Trait Self-Control Scale (Bertrams and Dickhäuser, 2009; English original: Tangney et al., 2004; α = 0.87), and the Perceived Stress Scale (Cohen et al., 1983; α = 0.81). Self-control capacity and trait self-control were highly correlated, *r* = 0.55, *p* < 0.001, and both self-control concepts were linked to lower stress (self-control capacity: *r* = −0.53, *p* < 0.001, trait self-control: *r* = −0.37, *p* < 0.001). However, self-control capacity predicted stress over and above trait self-control, β = −0.47, *p* < 0.001, whereas trait self-control did not predict stress over and above self-control capacity, β = −0.11, *p* = 0.35. Overall, the pretests suggest that the Self-Control Capacity Scale applied in the present study is a reliable and valid measure.
